# Red blood cell folate levels in Canadian Inuit women of childbearing years: influence of food security, body mass index, smoking, education, and vitamin use

**DOI:** 10.17269/s41997-018-0085-y

**Published:** 2018-05-09

**Authors:** Kait Duncan, Anders C. Erickson, Grace M. Egeland, Hope Weiler, Laura T. Arbour

**Affiliations:** 1 Department of Medicine, Island Medical Program, University of British Columbia, Vancouver, British Columbia Canada; 2 School of Population and Public Health, University of British Columbia, Vancouver, British Columbia Canada; 3 Department of Global Public Health and Primary Care, Bergen, Norway; 4 Division of Epidemiology, Norwegian Institute of Public Health, Bergen, Norway; 5 School of Human Nutrition, McGill University Macdonald Campus, Ste Anne de Bellevue, Quebec Canada; 6 Division of Medical Sciences, University of Victoria, Victoria, British Columbia Canada; 7 UBC Department of Medical Genetics, Island Medical Program, University of Victoria, Medical Sciences Building, Rm 104, 3800 Finnerty Rd, Victoria, British Columbia V8P 5C2 Canada

**Keywords:** Inuit, Indigenous, Folic acid, Red blood cell folate, Body mass index, Food security, Inuits, Autochtones, Acide folique, Folate érythrocytaire, Indice de masse corporelle, Sécurité alimentaire

## Abstract

**Background:**

The benefits of folic acid for prevention of congenital anomalies are well known. For the Inuit of Canada, where vitamin use is low and access to folate-rich foods limited, fortification is likely a major source of intake. We sought to determine whether red blood cell folate (RBCF) levels of Inuit women reached accepted target levels.

**Methods:**

The Inuit Health Survey, 2007–2008, included evaluation of RBCF levels among 249 randomly selected non-pregnant women of reproductive age. Using descriptive statistics and linear regression analyses, RBCF levels were assessed and compared across several socio-demographic variables to evaluate the characteristics associated with RBCF status.

**Results:**

Mean (SD) RBCF levels of 935.5 nmol/L (± 192) reached proposed target levels (> 906 nmol/L); however, 47% of women had lower than target levels. In bivariate analysis, non-smoking, higher education, higher income, food security, increased body mass index, and vitamin use were each significantly associated with higher RBCF. Increased levels of smoking had a negative association with RBCF levels (− 5.8 nmol/L per cigarette smoked per day (*p* = 0.001)). A total of 6.8% of women reported taking vitamin supplements, resulting in a 226 nmol/L higher RBCF level on average compared to non-users (*p* < 0.001).

**Conclusion:**

While mean levels of folate reached target levels, this was largely driven by the small number of women taking vitamin supplements. Our results suggest that folate status is often too low in Inuit women of childbearing years. Initiatives to improve food security, culturally relevant education on folate-rich traditional foods, vitamin supplements, and smoking cessation/reduction programs may benefit Inuit women and improve birth outcomes.

## Résumé

**Objectif:**

Les bienfaits de l’acide folique pour prévenir les anomalies congénitales sont connus. Pour les Inuits du Canada, qui ont une faible consommation de vitamines et un accès limité aux aliments riches en folates, les aliments enrichis sont probablement une importante source d’acide folique. Nous avons cherché à déterminer si les niveaux de folate érythrocytaire des femmes inuites atteignent les niveaux cibles acceptés.

**Méthode:**

L’Enquête de santé auprès des Inuits (2007–2008) a entre autres évalué les niveaux de folate érythrocytaire de 249 femmes non enceintes mais en âge de procréer sélectionnées au hasard. À l’aide de statistiques descriptives et d’analyses de régression linéaires, nous avons calculé et comparé les niveaux de folate érythrocytaire selon plusieurs variables sociodémographiques afin d’évaluer les caractéristiques associées au statut en folate érythrocytaire.

**Résultats:**

Les niveaux moyens (É.-T.) de folate érythrocytaire de 935,5 nmol/L (± 192) atteignaient les niveaux cibles proposés (> 906 nmol/L), mais 47% des femmes avaient des niveaux inférieurs à la cible. Selon les analyses bivariées, le fait de ne pas fumer, les études supérieures, le revenu élevé, la sécurité alimentaire, l’indice de masse corporelle élevé et la consommation de vitamines présentaient chacune une corrélation significative avec le folate érythrocytaire élevé. Les niveaux de tabagisme élevés présentaient une association négative avec les niveaux de folate érythrocytaire (− 5,8 nmol/L par cigarette fumée par jour (*p* = 0,001)). En tout, 6,8% des femmes disaient prendre des suppléments vitaminiques, et leurs niveaux de folate érythrocytaire étaient en moyenne plus élevés de 226 nmol/L que ceux des femmes ne prenant pas de suppléments vitaminiques (*p* < 0,001).

**Conclusion:**

Les niveaux moyens de folate atteignaient les niveaux cibles, mais ce résultat était en grande partie attribuable au petit nombre de femmes prenant des suppléments vitaminiques. Nos données indiquent que le statut en folate est souvent trop faible chez les femmes inuites en âge de procréer. Des initiatives d’amélioration de la sécurité alimentaire, une sensibilisation culturellement appropriée aux aliments traditionnels riches en folate, des suppléments vitaminiques et des programmes d’abandon ou de réduction du tabagisme pourraient être bénéfiques pour les femmes inuites et améliorer les issues de la grossesse.

## Introduction

An adequate maternal blood folate level during the periconceptional period significantly reduces the likelihood of a neural tube defect at birth (Czeizel and Dudas 1992). For this reason, Canada introduced mandatory folic acid fortification of cereal and grain products in November 1998 as a public health measure to reduce the incidence of neural tube defects (Public Health Agency of Canada 1998). In addition, there is increasing evidence that periconceptional folic acid intake also decreases the risk of other congenital malformations, including heart defects (Ionescu-Ittu et al. 2009; Czeizel et al. 2004; Czeizel et al. 2015). It has been previously observed that Inuit, residing in Canada’s Northern regions of Baffin Island, Nunavut and Nunavik, had nearly twice the rate of total birth defects compared to non-Inuit in other parts of the country (Arbour et al. 2004), increased rates which persist in more recent assessments, especially for congenital heart defects (Public Health Agency of Canada 2013). The potential protective effect of folic acid in the reduction of preterm births is also of growing interest (Chen et al. 2015) and is of relevance for Nunavut which has the highest rate of preterm births (12%) in the country (Mehaffey et al. 2010). Ninety-five percent of births in Nunavut are to Inuit women (Luo et al. 2010).

The traditional diet in Arctic regions is known to be high in fish, sea mammals, and meat, but low in plant food (Kuhnlein et al. 1996). Traditional food sources providing folate within Arctic regions include seaweed, tundra plants, caribou, moose, and ring seal liver (Hidiroglou et al. 2008); however, it has been shown that the modern Inuit derive their dietary folate nearly entirely from fortified market foods (Kuhnlein et al. 2004). Given the low baseline levels of folate in Inuit and other Northern population diets (Arbour et al. 2002; Moffatt 1995), it remains unclear whether such intakes are sufficient to reach target levels of red blood cell folate (RBCF) to prevent congenital anomalies. Daly et al. in their classic study suggest RBCF levels of 906 nmol/L can be reached with 200 mcg/day of folate through dietary intake and 400 mcg/day via a daily folic acid supplement (Daly et al. 1995). However, evidence shows that only a small proportion of Inuit women of childbearing years use multivitamins (Berti et al. 2008). This is compounded by the high prevalence of food insecurity in Canadian Inuit communities which has been noted to associate with lower dietary intake of key nutrients and other biomarkers of nutritional deficiency (Egeland et al. 2011). Furthermore, cigarette smoking is known to adversely influence folate levels (Oncel et al. 2012). Given that approximately 80% of pregnant Inuit women smoke cigarettes (Mehaffey et al. 2010), assessment of RBCF is pertinent. Thus, the primary objective of the current study was to determine whether the RBCF levels of Inuit women of childbearing age fall within the levels projected to prevent congenital anomalies and possibly reduce other adverse birth outcomes. The secondary objective was to evaluate the characteristics associated with RBCF levels among Inuit women. This analysis of RBCF status in women of childbearing years was part of the cross-sectional Inuit Health Survey (IHS) carried out in conjunction with the International Polar Year (IPY) Program of 2007–2008 (Saudny et al. 2012) which included 36 Inuit communities.

## Methods

### Community involvement and oversight

The cross-sectional International Polar Year Inuit Health Survey was undertaken to assess the overall health, wellness, and living conditions of Canada’s Inuit population residing in three land claim areas (Saudny et al. 2012). The study was developed under the direction of the IPY Steering Committee. The committee included representatives of Inuit organizations and community members from Nunavut, the Inuvialuit region of Northwest Territories (NWT), and Nunatsiavut, along with local and southern researchers, and government organizations. For the full list of the IPY Steering Committee, see http://www.mcgill.ca/cine/resources/ihs/steering-committees (McGill Centre for Indigenous Peoples’ Nutrition and Environment 2018). Scientific research license was received from the Nunavut Research Institute and the Aurora Research Institute (Inuvik, NWT). The Nunatsiavut review board waived the requirement for a license because the IHS team had engaged in an extensive participatory process.

The Steering Committee met on a regular basis to guide the content and methods, and to review the results of the Survey. Research agreements were put in place which considered the use and stewardship of samples and data. Our Inuit partners called for a broad survey and the idea for this specific project resulting in this paper was reviewed by the Steering Committee. Their comments and ideas were incorporated into the work plan. Our drafts and final manuscript were reviewed by the National Inuit Health Surveys Working Group of Inuit Tapiriit Kanatami who contributed to and approved the content of the final manuscript.

### Consent

In support of Inuit oral traditions, a “visual” consent form was created as a DVD in relevant Inuit languages (Inuktitut, Inuinnaqtun, Siglitin, Uummarmiutun, Nattilik, and Inutitut). The DVD depicted the written consent form word-for-word, including all clinical and laboratory procedures. After watching the DVD, participants consenting to participation signed the written consent form (Saudny et al. 2012).

### Data collection

Of a total of 2796 invited households, 1901 participated, with a total enrollment of 2595 adults. Pregnant women were excluded. Participants completed questionnaires, had medical and anthropometric measurements taken, and had fasting venous blood samples drawn. As the majority of communities are coastal, the research was supported by the Canadian Coast Guard Ship Amundsen which housed centrifuges and − 80 °C freezers for the processing of blood specimens. Of the participants, 249 randomly selected women of childbearing years (between the ages of 18 and 39) were included in the RBCF substudy, with 192 from Nunavut, 23 from Nunatsiavut, and 34 from the Inuvialuit Settlement Region (ISR) (Saudny et al. 2012). Survey data collected of relevance to the current study included age (years), the use of folate-containing supplements and/or multivitamins (yes vs. no), anthropometry (height and weight for calculating body mass index, BMI, kg/m^2^), waist circumference and percent body fat using leg-to-leg bioelectrical impedance instrument analysis (Tanita TBF-300GS, Arlington Heights, IL, USA), present and past smoking habits, household food security assessments (food secure, moderate food insecurity, and severe food insecurity) (Egeland et al. 2011), education, and income (Saudny et al. 2012). To ensure accuracy, participants were asked to bring their folic acid and other vitamin supplements to the interview. All folic acid supplements and multivitamins containing folic acid were included and are referred to as “vitamins.”

### Red blood cell folate

Analysis of blood sample collections included RBCF levels on the 249 women for this substudy (Saudny et al. 2012). Blood was collected in EDTA containing tubes and protected from light. Hematocrit was determined as per standard protocol. For RBCF, 50 μl of sample was added to 1.0 ml of folate ascorbic acid in a separate tube, followed by mixing and allowing the hemolysate to separate. The samples were then frozen at − 20 °C and shipped frozen to Nutrasource Diagnostics, Guelph, ON, where they were stored at − 80 °C until analysis by Quest Diagnostics, San Juan Capistrano, CA.

### Quality control

All Quest Diagnostics’ testing locations are subject to Clinical Laboratory Improvement Amendments of 1988 (CLIA-88) certification and maintain current CLIA licenses. Quest Diagnostics’ main laboratories are accredited by the College of American Pathologists (CAP).

### Statistical analysis

Descriptive statistics and bivariate (simple) linear regression analyses were used to evaluate the relationships between RBCF levels (nmol/L) and the aforementioned characteristics (Table 1). Pair-wise correlation tests were performed to show the inter-relationships between the variables (Table 2). Sensitivity analyses restricted to non-supplement users were carried out to evaluate consistency in results. All statistical analyses were conducted using Stata 11-IC.

**Table 1:** Red blood cell folate levels (nmol/L) by demographic characteristics (*N* = 249)

Independent variables: categorical	Frequency, *N* (%)	RBC folateª Mean (sd)	*β* (95% CI)§	*p* value
Current smoker				
No	46 (18.5)	1005.6 (217.0)	Ref	
Yes	203 (81.5)	919.6 (182.6)	− 86.2 (−147.1–− 25.3)	0.01
Food security				
Secure	77 (30.9)	964.6 (190.5)	Ref	
Moderate insecure	84 (34.5)	933.4 (194.9)	− 34.2 (−94.0–25.7)	0.26
Severe insecure	76 (30.5)	901.9 (185.0)	− 62.0 (−123.9–− .004)	0.05
Missing	10 (4.0)	984.4 (212.6)	–	–
Income				
< $20,000	134 (53.8)	920.7 (187.4)	Ref	
$20,000–$39,999	36 (14.5)	938.7 (199.4)	18.0 (−52.6–88.6)	0.62
$40,000–$59,999	20 (8.0)	991.5 (209.4)	70.8 (−19.3–160.9)	0.12
> $60,000	21 (8.4)	1015.3 (201.6)	94.5 (6.3–182.8)	0.04
Did not report/missing	38 (15.3)	911.1 (178.5)	–	–
Education				
Primary	39 (15.7)	883.2 (183.9)	Ref	
Some secondary	98 (39.4)	919.1 (183.5)	36.0 (−34.7–106.6)	0.32
Completed secondary	58 (23.3)	959.2 (177.2)	76.0 (−1.3–153.3)	0.05
College/university	46 (18.5)	984.9 (219.9)	101.8 (20.5–183.0)	0.01
Missing	8 (3.2)	935.4 (220.9)	–	–
Vitamin use				
No	232 (93.2)	920.1 (181.4)	Ref	
Yes	17 (6.8)	1146.1 (212.8)	226.0 (135.2–316.9)	< 0.001
Continuous	Min-max	Mean (sd)		
Age (years)	18–39	29.1 (6.0)	− 1.2 (−5.2–2.8)	0.55
Cigarettes/day	0–40	7.7 (6.7)	− 5.8 (−9.3–− 2.3)	0.001
Years smoked*	0–29	13.3 (7.5)	− 4.7 (−7.9–− 1.6)	< 0.01
Body mass index (BMI)	17.3–58.3	28.5 (6.7)	4.4 (0.8–8.0)	0.02
Waist circumference (cm)	61–156.5	92.7 (16.8)	2.0 (0.6–3.4)	0.007
Body fat (%)	10.5–54.8	33.4 (9.8)	3.7 (1.2–6.2)	0.004

^a^Red blood cell folate measured in nmol/L, range 373.7–1440.5, mean 935.5 nmol/L (SD 191.9); vitamin use includes multivitamin use with folic acid and folic acid supplements

§β coefficients associated with each independent variable obtained from unadjusted linear regression with RBCF as dependent variable

*Years smoked includes former smokers

**Table 2:** Unadjusted correlation coefficients between study variables

	RBCF	Age	Cigs/day	Years smoked	BMI	Education	Food insecurity	Income	Vitamin use
RBCF	1.0								
Age (years)	− 0.038	1.0							
Cigarettes/day	− 0.202**	0.053	1.0						
Years smoked	− 0.184*	0.645**	0.343**	1.0					
BMI (kg/m^2^)	0.153*	0.081	− 0.141*	− 0.045	1.0				
Education	0.177*	0.007	− 0.255**	− 0.186*	0.148*	1.0			
Food insecure	− 0.131*	0.131*	0.113	0.234**	− 0.172*	− 0.335**	1.0		
Income	0.165*	0.263**	− 0.178*	0.039	0.234**	0.371**	− 0.367**	1.0	
Vitamin use	0.298**	0.034	− 0.057	− 0.058	− 0.123	0.185*	− 0.145*	0.152*	1.0

Coefficients are derived from pair-wise correlation tests

RBCF: red blood cell folate (nmol/L); age: increasing years; cigarettes/day: daily number of cigarettes smoked per day for current smokers; years smoked: includes former smokers; BMI: body mass index; education: categorical 1 to 4 where 1 = primary and 4 = post-secondary; food insecurity: categorical 1 to 3 where 1 = food secure, 2 = moderate insecure, 3 = severe insecure; income: categorical 1 to 4 where 1 = < $20,000/year and 4 = > $60,000/year Canadian dollars

*Significant at *p* = 0.05

**Significant at *p* = 0.05 using Bonferroni correction for multiple testing

## Results

Here, we highlight results from the descriptive and bivariate analyses, all of which are presented in Table 1. The average age of women participating in this substudy was 29.1 ± 6 years (Table 1). Eighty-two percent were current smokers, 35% of whom smoked 10 or more cigarettes per day. Vitamin use was reported by 6.8%. Sixty-five percent reported moderate or severe food insecurity. Forty-two percent had completed high school or had higher education. These descriptive statistics are similar to that of the overall study population from which the random sample was drawn.

**Fig. 1:**
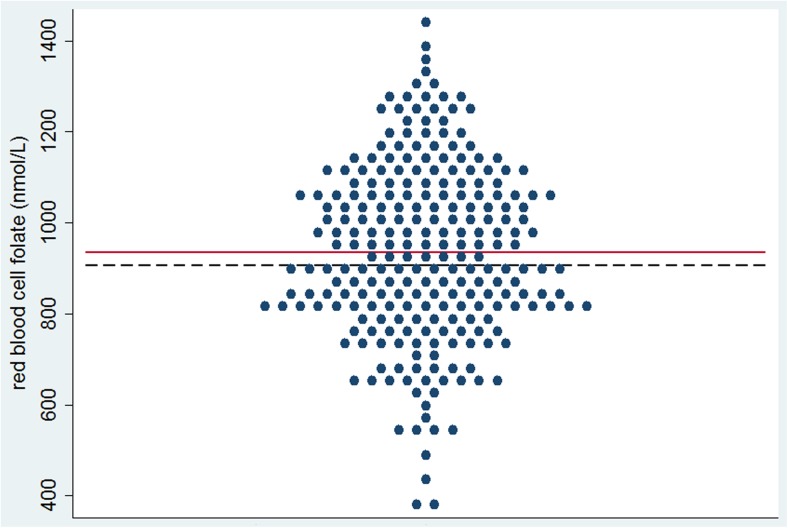
Scatter plot of red blood cell folate values from Inuit women of childbearing age across all three sample sites. Solid red line denotes population mean (935.5 ± 192 nmol/L); dashed black line denotes target reference (906 nmol/L). Forty-seven percent were below the target

The mean RBCF value was 935.5 ± 192 nmol/L (range 373.7 to 1440.5 nmol/L), indicating considerable variability in values (Fig. 1). The mean RBCF level of non-vitamin users was significantly lower than that of the vitamin users (920.1 ± 181.4 vs. 1146.1 ± 212.8 nmol/L, *p* < 0.001).

In the bivariate linear regression analyses (Table 1), all smoking-related variables had a statistically significant negative relationship with RBCF levels, with a 5.8 nmol/L lower RBCF level for every additional cigarette smoked per day (*p* = 0.001) and 4.7 nmol/L lower RBCF level for every additional year smoked (*p* < 0.01).

There was a clear trend of increasing RBCF levels with increased levels of income and education. Similarly, there was a 62.0 nmol/L (*p* = 0.05) higher RBCF level in food-secure participants compared with those with severe food insecurity. There was a statistically significant positive association between RBCF and BMI (4.4 nmol/L per unit BMI, *p* = 0.02), with similar effects observed with waist circumference and percent body fat.

Sensitivity analysis restricted to non-vitamin users shows similar associations, with the exception of weaker and non-significant associations between RBCF, education, and food security. Additional sensitivity analyses reveal that the 10 participants missing the food (in)security data were more similar to the food-secure group in terms of RBCF levels (984.4 ± 212.6 nmol/L), higher education, and income (Table 1). The inter-relationship between the characteristics was measured using pair-wise correlation analyses (Table 2), which show that higher income, education, and BMI are all positively correlated with food security and RBFC levels.

## Discussion

Folate is a key factor in DNA biosynthesis and cell division (Scott 1999). The knowledge that folate has an important role in reducing birth defects and improving birth outcomes has evolved and strengthened over the last 30 years (Czeizel et al. 2015; Chen et al. 2015). The often-quoted target of RBCF concentration of 906 nmol/L is based on a dose-response curve by Daly et al. assessing optimum levels of folic acid supplementation and fortification (on the background of assumed levels of daily folate intake) to prevent congenital anomalies, specifically neural tube defects (Daly et al. 1995). With a measured mean RBCF level of 935.5 ± 192 nmol/L, our findings suggest that at the population level, the average Inuit woman of childbearing age has a sufficient folate/folic acid intake to reach target levels. Although this is reassuring, it is concerning that 47% of women were below that target. Of note, fewer than 7% of the women in this study were using vitamin supplements (mean RBCF level of 920.1 ± 181.4 nmol/L).

The RBCF levels are similar to our previous case-control study of congenital heart malformations, which included 76 Inuit women of childbearing age from Nunavut, whose mean RBCF level was found to be 947.0 ± 32.0 nmol/L (Arbour et al. 2007). It is notable that none of the participants were taking vitamins at the time of that study (2003–2004). Mandated fortification together with high bioavailability of synthetic folic acid is likely responsible for the reasonable RBCF levels demonstrated in our results. Given the background of a low-folate diet (Arbour et al. 2002; Moffatt 1995), these results may reflect a greater intake of fortified market food, which is relatively low cost and nutrient-poor compared to traditional foods (Kuhnlein et al. 2004; Kuhnlein and Receveur 2007). Although some traditional food sources contain folate (Hidiroglou et al. 2008) (see Table 3), the modern day Inuit derive nearly all their dietary folate from fortified food. An analysis of post-fortification dietary intake carried out by Kuhnlein and Receveur found that traditional food now comprises only 17–28% of the average daily energy intake of adults in the Northern communities studied, whereas white bread and biscuits (items subject to folic acid fortification) were the third and fourth most commonly consumed market food item by weight after tea and sugar (Kuhnlein and Receveur 2007). Such a grain-based diet is associated with lower income and lower educational attainment elsewhere in Canada (Tarasuk et al. 2010) and may also be relevant in Nunavut (Zienczuk and Egeland 2012; Huet et al. 2012). Of relevance, for those considering pregnancy or in the early weeks of pregnancy, some liver sources of high folate can also contain high levels of retinol (Egeland et al. 2004) and methyl mercury (Laird et al. 2013), which, when consumed in excess, can be harmful to the developing fetus. For example, it is suggested that if ring seal liver is eaten, servings be limited to less than 50 g during early pregnancy or when there is a possibility of pregnancy (Egeland et al. 2004) or be replaced by other nutrient-rich traditional food (Laird et al. 2013).

**Table 3:** Traditional food source of folate

Food source	Total folate (μg/100 g)
Ring seal liver	1003 ± 218
Walrus liver	925 ± 128
Seaweed	447
Caribou liver	374 ± 152
Moose liver	268
Eggs of Cisco	250
Caribou kidney	72.6
Clam flesh	56.8

Derived from Hidiroglou et al. 2008

To put our results into the Canadian perspective, we can compare our results to the Canadian Health Measures Survey (CHMS), a representative sample of more than 5000 Canadians, of whom 644 were women of reproductive age (20–39), that was conducted over a similar time period as the current study (2007–2009) (Statistics Canada 2010a). The average RBCF of Inuit women in the current study was notably less than that observed among women of childbearing years in the CHMS (935.5 ± 192 vs 1279.0 ± 50.9 nmol/L). Importantly, 47% of Inuit women had RBCF levels below target (Fig. 1), which was more than double (22%) that in the CHMS (Statistics Canada 2010a). Interestingly, Inuit women who reported taking vitamin supplements with folate at the time of the study (*n* = 17) had mean RBCF levels similar to those of the 644 women aged 20–39 in the CHMS (1146.1 ± 212.8 vs 1279.0 ± 50.9 nmol/L, respectively).

It is well accepted that even in affluent nations, socio-economic inequality is associated with decreased diet and nutritional quality in the lower socio-economic tiers (Mullie et al. 2010). Using the 2004 Canadian Community Health Survey (CCHS), Tarasuk et al. (2010) found that women (19–50 years) with lower income and education had a higher prevalence of folate deficiency in comparison to similarly aged women of higher socio-economic status. The same trend has been seen in the US, where ethnicity and low income status have consistently been predictors of low blood folate (Yang et al. 2007), factors relevant to the Inuit women in our study (Huet et al. 2012; Statistics Canada 2010b; Egeland 2010). Only a third of the women in our study were from households considered “food secure,” which showed significantly higher overall RBCF levels compared to those reporting moderate or severe food insecurity. Our findings show a positive correlation among BMI, food security, higher income, and education, which has been previously observed in a broader analysis of the IHS, but that study did not include RBCF (Zienczuk and Egeland 2012). Of interest is the associated higher RBCF with greater BMI. Our results are consistent with recent evidence that RBCF is higher among obese people; however, serum concentrations of folate may not necessarily be higher (Bird et al. 2015).

Cigarette smoking reduces RBCF stores and distribution (Oncel et al. 2012). Supporting our findings of negative association of smoking and RBCF, evidence was also seen in the third US National Health and Nutrition Examination Survey where significantly lower RBCF levels were shown in individuals with high smoke exposure (smokers and passive smokers), even after adjusting for dietary folate intake (Mannino et al. 2003). Given that 81.5% of the participants in our study reported smoking, smoking status remains an important consideration regarding poor RBCF status.

Of our study’s 249 participants, only 6.8% were taking supplements, in contrast to 47% in a 2004 survey of 20,263 non-pregnant North American women between the ages of 18 and 44 (Sullivan et al. 2009). As determined by the 2006/2007 Canadian Maternity Experiences survey (Public Health Agency of Canada 2009) of 8542 women, 57.7% (95% CI 56.4–59.0) took folate periconceptionally (1 month prior to pregnancy and continuing for 3 months into pregnancy). However, only 13.6% of women living in Nunavut reported taking supplements in the periconceptional period. Of further relevance, Berti et al. (2008) reviewed nutrient intake and vitamin use recorded in dietary studies between 1987 and 1999 in 1300 non-pregnant and non-lactating Arctic women of childbearing years and found that only 5% were taking multivitamins.

Risk factors for low supplement use include low income and educational achievement (Public Health Agency of Canada 2009; Colapinto et al. 2011). Even in countries with well-developed folic acid promotion and monitoring programs, such as the Netherlands, there is a significant disparity in supplement usage among the highest and lowest socio-economic groups (59% vs. 22%, respectively) (de Jong-van den Berg 2008). In our study, vitamin use had a significant positive impact on RBCF levels, with a more than 200 nmol/L higher value among vitamin users.

This evidence underscores the importance of recognizing those women who would benefit most from supplemental folic acid fortification and other programs to improve folate status. Given organogenesis occurs in the first weeks of pregnancy, and few pregnancies are planned, current recommendations are that all women who could become pregnant take a daily multivitamin containing folic acid (Health Canada 2018). Smoking and food insecurity are determinants of reduced folate status; therefore, with increased population prevalence of both, the majority of Inuit women would benefit by health promotion in these areas. Programs to reduce smoking (https://nuquits.gov.nu.ca/) and improve food security (https://itk.ca/nuluaq-mapping-project/about/) are currently underway. Integration of education on the use of traditional foods with folate, and improved access to folic acid supplements, are interventions that could improve RBCF status and lower risks for adverse birth outcomes.

### Limitations

This was a cross-sectional study with a limited sample size.

## Conclusion

The Inuit Health Survey provided the setting to evaluate the blood folate status of Inuit women of childbearing years, an issue of ongoing concern. The findings of our study are striking in that nearly half of Inuit women do not reach target levels of RBCF, and that lower folate status associates with low income, low education, food insecurity, and smoking. Our results indicate that ongoing health promotion of the benefits of supplemental vitamin use for women of childbearing years should be undertaken, alongside programs to reduce food insecurity and smoking in pregnant women.
